# Acute Disseminated Encephalomyelitis: A rare form of COVID-19's neurotropism

**DOI:** 10.1016/j.amsu.2021.102940

**Published:** 2021-10-11

**Authors:** Samia Berrichi, Zakaria Bouayed, Sara Berrajaa, Choukri Bahouh, Amine Mohammed Oulalite, Badie Douqchi, Islam Bella, Houssam Bkiyar, Brahim Housni

**Affiliations:** a Department of Anesthesiology, Intensive Care Medicine and Resuscitation, MOHAMMED VI University Hospital Center, Oujda, Morocco; b Simulation Center, Faculty of Medicine and Pharmacy, Oujda, Morocco

**Keywords:** COVID-19, Neurotropism, ADEM

## Abstract

**Introduction:**

the COVID-19 pandemic still accounts for thousands of cases every day. It's neurological involvement has been well documented most likely due to auto-immune mechanisms than the virus itself.

**Case report:**

we report the case of a 38 years old women who developed an Acute Disseminated Encephalomyelitis following a COVID-19 infection, with a favorable outcome after immunosuppressive therapy.

**Discussion:**

In this chapter, we discuss ADEM's pathogenesis as well as its clinical and radiological features before detailing its relationship with infectious and vaccination episodes. We also discuss how our patient disease evolved.

**Conclusion:**

Acute Disseminated Encephalomyelitis is an immune-mediated disorder in which the widespread inflammation of the brain and spinal cord is responsible for a variety of symptoms. The novel COVID-19 virus and its vaccine are both a newly incriminated etiologies of this demyelinating disorder.

## Highlights

• COVID-19 neurotropism has been well established and its neurological manifestations are varied.
• Demyelinating disorders are immune-induced disease most likely due to an antigenic analogy between the virus and myelin constituents.
• COVID-19 induced ADEM is extremely rare, only a dozen cases have been reported worldwide, and ours is the only case reported in our country.

## 1 Introduction

The novel SARS-Cov-2 virus responsible of the current COVID-19 pandemic still raises a sustained interest fueling more studies every day.

The neurotropism of the SARS-Cov-2 has been well documented in the literature and a wide range of neurological disorders are being reported every day.

We report the case of a 38 years old women hospitalized in our unit for the management of a monophasic Acute Disseminated Encephalomyelitis (ADEM) following a recent COVID-19 infection.

## 2 Case report

A 38 years old women, without any prior medical history first developed a cough and a fever 10 days prior to her admission for which she received a symptomatic treatment by a local physician without any improvement. 3 days later the patient developed a headache and an incoherent speech associated to behavioral changes (aggressiveness), adding to that visual and auditory hallucinations as well as a rapidly progressive motor deficit of the lower limbs.

In light of the patient's neurological symptoms and the persistence of her respiratory signs, she was admitted to the ER. Initial physical examination revealed an agitated and confused patient with a GCS of 13/15, afebrile (36.9 °C), with an oxygen saturation of 88% on ambient air and 95% under High Flow Therapy (70% FiO2 at a 60L/min flow rate). Neurological examination revealed a lower limb flaccid paraparesis, a paresthesia with an umbilicus sensory level, as well as a urinary retention.

Laboratory findings showed an elevated WBC (22 × 10 [3]/μL), predominantly neutrophils (93%), a low lymphocytes count (750/μL), a CRP level of 152,61 mg/L, with a hemoglobin at 11 g/dl, serum ferritin level of 1050,32 ng/mL, and an IL-6 level of 70pg/mL. normal electrolyte levels, as well as normal liver and kidney function tests. Arterial blood gas tests revealed a pH of 7.34, a PaCO2 of 34.4 mmHg and a PaO2 of 68 mmHg, a nasal swap was performed and the SARS-Cov-2 RT-PCR came back positive, CSF study following a lumbar punctation revealed a high WCC (17 cell/mm^3^), positive oligoclonal band test (OCB+) and IgG index (1.2).

**Fig. 1:**
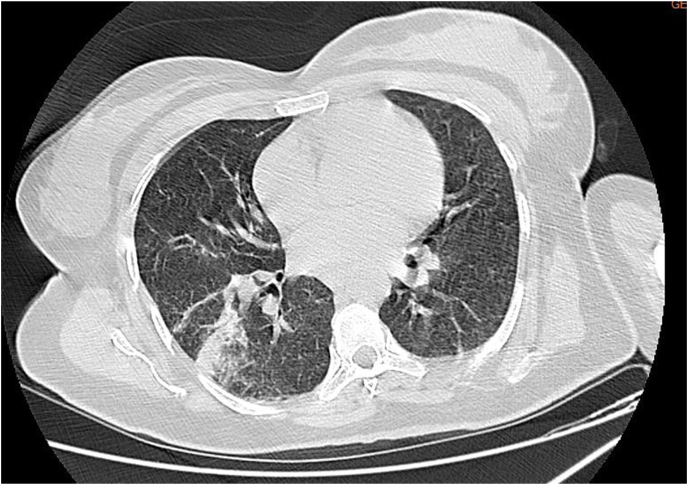
Axial lung window showing ground-glass opacities with interlobular septal thickening realizing a crazy paving pattern, as well as right lung peripheral pulmonary consolidation. SARS-Cov-2 RT-PCR positive, CT findings consistent with COVID-19 pneumonia CO-RADS 6.

**Fig. 2:**
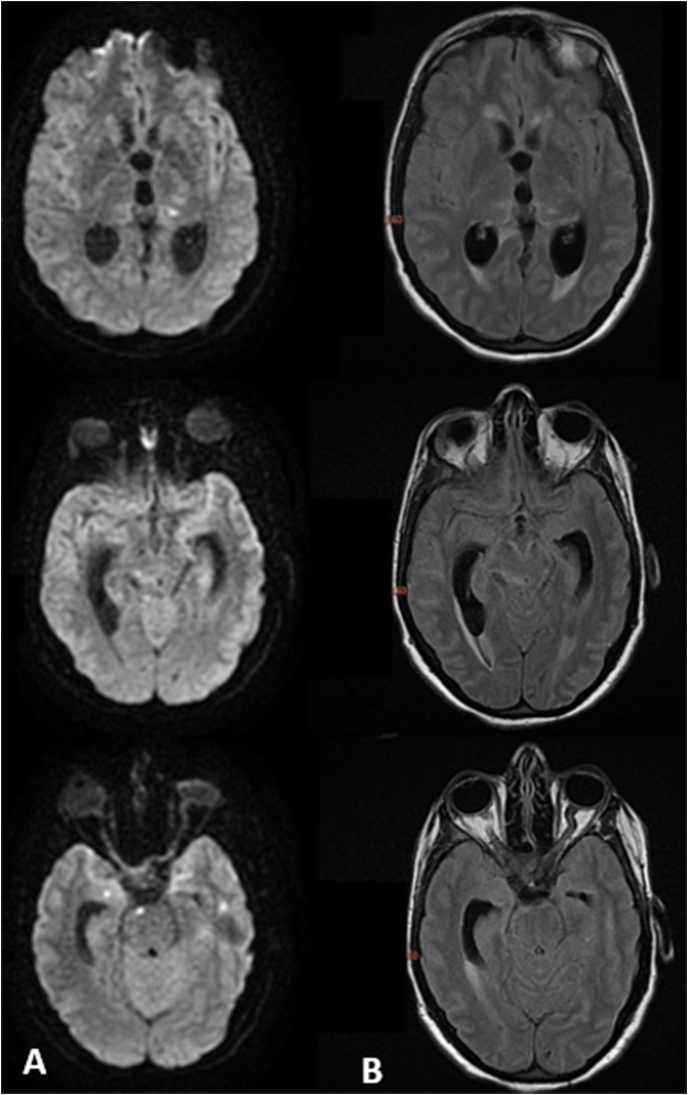
Axial Fluid-attenuated inversion recovery (B) and Diffusion-weighted imaging (A) sequence images showing multiple nodular hyperintensities, varying in size, located in the juxtacortical white matter, the left thalamus, and the brainstem.

**Fig. 3:**
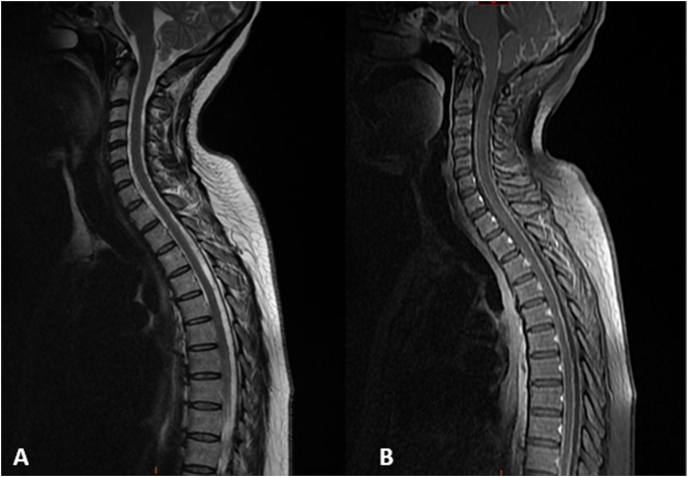
Sagittal T2 (A) and T1 C+ (B) spine MRI showing discrete T2 hyperintensities with contrast enhancement along the posterior column of the cervical spinal cord.

Chest CT showed abnormalities consistent with a COVID-19 pneumonia involving overall around 25–50% of the lungs. (Fig. 1). MRI of the brain (Fig. 2) revealed FLAIR and DWI nodular hyperintensities in the juxtacortical frontal and temporal white matter, as well as the subcortical grey matter, specifically the left thalamus and the brainstem. While the spine MRI (Fig. 3) showed discrete T2 hyperintensities with contrast enhancement along the posterior column of the cervical spinal cord, suggestive of myelitis.

Given the clinical, biological and radiological features of the disease, and after concertation, we retained the diagnosis of Acute Disseminated Encephalomyelitis (ADEM).

The patient was treated with Ceftriaxone, Ciprofloxacin and Acyclovir 3 days prior to an intravenous injection of 400mg of Tocilizumab, and high doses of methylprednisolone.

The patient's neurological symptoms slowly regressed within 5 days, and a progressive withdrawal from oxygen was performed simultaneously.

Follow-up laboratory tests showed an improvement of the inflammatory markers.

The patient was discharged within 11 days after admission.

## 3 Discussion

Neurological features related to SARS-Cov-2 have been wildly reported [1] and the neurotropism of the SARS-Cov-2 virus much as the other coronaviruses has been firmly established [2,3].

We report the case of a patient who developed an Acute Disseminated Encephalomyelitis (ADEM) following a SARS-Cov-2 infection.

ADEM is an acute inflammatory demyelinating disorder of the central nervous system (CNS) most commonly affecting children [4,5], typically triggered by a viral or bacterial infection (often of the upper respiratory tract) or post vaccination [6]. Relatively rare [5], it's diagnosis often raises the issue of differential diagnosis with other causes of acquired CNS demyelinating syndromes [4,7], to that end, consensus clinical diagnostical criteria have been proposed [8] easing the process of a positive diagnosis.

ADEM's pathogenesis isn't fully understood, the leading theory suggests an antigenic analogy between myelin antigens and the pathogen responsible for ADEM [9], which explains why cases of ADEM have been reported both in patients with COVID-19 and subjects vaccinated with the AZD1222 COVID-19 vaccine [10,11]. In fact, a literature review by Sriwastava et Al [12] identified a total of 43 cases of COVID-19-related myelitis 10 of which being ADEM cases.

Typically, ADEM is preceded (days and up to few weeks prior), by an infectious episode or a vaccination [13]. Clinically, ADEM usually causes a monophasic demyelinating episode responsible of an acute onset of neurological symptoms depending on which region of SNC is affected, accompanied with encephalopathy, often rapidly deteriorating [13].

ADEM's radiological features may vary from punctate to sizable lesions, affecting the periventricular and subcortical white matter, as well as the grey matter, including the cortex, basal ganglia, and thalamus, also, infratentorial involvement of the brainstem, the cerebellum, and/or the spinal cord is also possible [7,[14], [15], [16]]. Keeping in mind that new lesions and/or enlargement of existing MRI lesions is possible throughout ADEM's evolution [15,17].

The patient was treated with immunosuppressive therapy using an IL-6 inhibitor (Tocilizumab) and high doses of methylprednisolone after an antibiotic coverage. The patient showed clinical and biological improvement within days. She was discharged with minimal residual symptoms (weakness of the lower limb) and put under gradually lower doses of oral prednisolone. No follow-up MRI was performed per patient's choice.

Our case report joins only a dozen cases of COVID-19 induced ADEM [12,18] that have been documented in the literature so far. In fact among the 525443 confirmed cases in Morrocco registered to this day [19], to the best of our knowledge no other cases of ADEM have been reported.

Although irrelevant to the subject at hand our case also shines light on a crucial key point in the management of COVID which is the importance of preventing any delay in the diagnosis of COVID-19 thus testing for the virus early-on which wasn't the case here as the patient only received a symptomatic treatment initially.

## 4 Conclusion

COVID-19 neurotropism is now well established, and more and more cases of COVID-19's neurological involvement are being reported every day.

Demyelinating disorders related to COVID-19 infections or vaccines constitute a rare neurological entity. The most common cases being of Acute Transverse Myelitis (ATM), ADEM cases constitute even a rarer entity.

This work has been reported in line with the SCARE 2020 Guidelines [20].

## Provenance and peer review

Not commissioned, externally peer-reviewed.

## Ethical approval

This is a case report, therefore Ethics committee/IRB approval is not required.

## Sources of funding for your research

This article hasn't received any funding whatsoever.

## Author contribution

SAMIA BERRICHI: Study conception, Data collection; data analysis; writing & editing.

ZAKARIA BOUAYED: Data collection; data analysis; writing & editing.

SARA BERRAJAA: Contributor.

CHOUKRI BAHOUH: Contributor.

AMINE MOHAMMED OULALITE: Contributor.

BADIE DOUQCHI: Contributor.

ISLAM BELLA: Contributor.

HOUSSAM BKIYAR: Supervision and review data validation.

HOUSNI BRAHIM: Supervision and review data validation.

## Registration of research studies

Name of the registry:
Unique Identifying number or registration ID:
Hyperlink to your specific registration (must be publicly accessible and will be checked):

## Consent

Written informed consent was obtained from the patient for publication of this case report and accompanying images. A copy of the written consent is available for review by the Editor-in-Chief of this journal on request.

## Guarantor

SAMIA BERRICHI.

ZAKARIA BOUAYED.

## Declaration of competing interest

There are no conflicts of interest.
